# Anti-cancer and antimicrobial potential of five soil *Streptomycetes*: a metabolomics-based study

**DOI:** 10.1098/rsos.211509

**Published:** 2022-02-09

**Authors:** Nada Osama, Walid Bakeer, Mai Raslan, Hanan A. Soliman, Usama Ramadan Abdelmohsen, Mohamed Sebak

**Affiliations:** ^1^ Biotechnology and Life Sciences Department, Faculty of Postgraduate Studies for Advanced Sciences, Beni-Suef University, Beni-Suef, 62511, Egypt; ^2^ Microbiology and Immunology Department, Faculty of Pharmacy, Beni-Suef University, Beni-Suef, 62511, Egypt; ^3^ Biochemistry Division, Chemistry Department, Faculty of Science, Beni-Suef University, Beni-Suef 62511, Egypt; ^4^ Department of Pharmacognosy, Faculty of Pharmacy, Minia University, Minia 61519, Egypt; ^5^ Department of Pharmacognosy, Faculty of Pharmacy, Deraya University, New Minia 61111, Egypt

**Keywords:** actinomycetes, anti-cancer, antimicrobial, dereplication, metabolomics, natural products

## Abstract

Lack of new anti-cancer and anti-infective agents directed the pharmaceutical research to natural products' discovery especially from actinomycetes as one of the major sources of bioactive compounds. Metabolomics- and dereplication-guided approach has been used successfully in chemical profiling of bioactive actinomycetes. We aimed to study the metabolomic profile of five bioactive actinomycetes to investigate the interesting metabolites responsible for their antimicrobial and anti-cancer activities. Three actinomycetes, namely, *Streptomyces* sp. SH8, SH10 and SH13, were found to exhibit broad spectrum of antimicrobial activities, whereas isolate SH4 showed the broadest antimicrobial activity against all tested strains. In addition, isolates SH8, SH10 and SH12 displayed potent cytotoxicity against the breast cancer cell line Michigan Cancer Foundation-7 (MCF-7), whereas isolates SH4 and SH12 exhibited potent anti-cancer activity against the hepatoma cell line hepatoma G2 (HepG2) compared with their weak inhibitory properties on the normal breast cells MCF-10A and normal liver cells transformed human liver epithelial-2 (THLE2), respectively. All bioactive isolates were molecularly identified as *Streptomyces* sp. via 16S rRNA gene sequencing. Our actinobacterial dereplication analysis revealed putative identification of several bioactive metabolites including tetracycline, oxytetracycline and a macrolide antibiotic, novamethymycin. Together, chemical profiling of bioactive *Streptomycetes* via dereplication and metabolomics helped in assigning their unique metabolites and predicting the bioactive compounds instigating their diverse bioactivities.

## 1. Introduction

Various natural product sources have significantly contributed to medication discovery and development especially in the treatment of different cancers and infections [1,2]. More than 40% of the drugs approved as anti-infective or anti-cancer agents in the period from 1981 to 2014 were based on either natural products or their derivatives [2]. However, the chemical complexity of the bioactive secondary metabolites as well as supply problems from their biological sources slowed down the pharmaceutical research in the area of natural products’ discovery in comparison with synthetic drugs [1]. However, the number of reports of antibiotic-resistant microorganisms in both community and clinical settings is growing with increasing rate of mortality. This increase in number of multi-drug resistant pathogens is the main challenge researchers facing to discover new antimicrobial agents with efficacy against those organisms [3]. Therefore, there is an increasing interest in discovering new natural products to combat the antibiotic resistance [4].

Interestingly, around half of the reported microbial natural products were isolated from actinomycetes [5], while *Streptomycetes* were reported to produce approximately 75% of all actinobacterial secondary metabolites [6,7]. Antimicrobial agents isolated from actinomycetes include streptomycin, rifamycin and gentamycin, whereas anti-cancer agents comprise mitomycin, aclarubicin, doxorubicin, mithramycin, neocarzinostatin and carzinophilin [2,8–10]. In actinomycetes, polyketide synthases and non-ribosomal peptide synthetases stimulate the production of secondary metabolites through different metabolic pathways [6].

Metabolomics is one of the most popular and wide-ranging applications of bioinformatics, which helps understand the primary and secondary metabolism of microorganisms, plants and animals [11]. Metabolomics particularly studies all the secondary metabolites produced by a biological system [12]. In addition, dereplication is one of the most popular tools used for separating new compounds and quickly identifying secondary metabolites isolated from microbial extracts based on reported secondary metabolites in the database such as Dictionary of Natural Products (DNPs) and AntiMarin database. Therefore, dereplication studies, in addition to multivariate data analysis (MVA), are commonly used in the drug discovery programmes [11,13].

Several previous studies have employed a bioassay-guided approach for the discovery of microbial natural products in Egypt [4,14], whereas only few studies used a bioassay- and metabolomics-guided approach for the chemical profiling of bioactive actinomycetes as well as isolation of new biologically active secondary metabolites from them [15]. In the current study, we aimed to conduct chemical profiling of some soil actinomycetes which have broad antimicrobial and anti-cancer activities using a dereplication- and metabolomics-guided approach to investigate the interesting secondary metabolites responsible for isolates' bioactivities and to assign the promising metabolites which could be targeted for isolation.

## 2. Experimental

### 2.1. Isolation of actinomycetes

Five actinomycetes were recovered from soil samples collected during the winter of 2016 from the Sherif-Pasha village in Beni-Suef Governorate, Egypt. The soil samples were collected from the top layer of agricultural soil and placed in sterile plastic bags and then transported to the laboratory for isolation. The actinobacterial isolates were isolated using a slightly modified soil serial dilution approach [16,17]. In brief, 1 g of each soil sample was diluted in 9 ml of 0.9% saline and then serially diluted with saline up to 1 × 10^−6^. Then, all tubes were mixed well using a rotatory shaker at 200 r.p.m. for 15 min. Subsequently, 1 ml from all diluted tubes was spread on starch casein agar containing nystatin (50 µg ml^−1^) to prevent fungal contamination and rifampicin (5 µg ml^−1^) to prevent bacterial contamination. Then, all inoculated plates were incubated for 7 days at 30°C. Actinomycete colonies were then characterized according to their morphology and pigmentation and then selected for further purification. Finally, pure colonies of actinomycetes were sub-cultured on starch casein agar and incubated at 30°C for 7 days, followed by storage at −80°C in 30% glycerol broth.

### 2.2. Antimicrobial activity screening

The antimicrobial activity of actinobacterial isolates was tested against some indicator strains, including the Gram-positive bacteria (*Staphylococcus aureus* (ATCC 43300), *Listeria monocytogenes* (ATCC 7644) and *Bacillus subtilis* (environmental sample)) and the Gram-negative bacteria (*Escherichia coli* (clinical isolate) and *Salmonella enterica* (ATCC 14028)) in addition to yeast (*Candida albicans* (ATCC 60193)) as previously described [18]. Briefly, each actinomycete was cultivated on International Streptomyces 2 Project (ISP2) agar at 30°C for 7 days. Then, 7 mm agar discs from actinobacterial growth of each isolate were removed and transferred to the surface of tryptone soya agar plates that had already been inoculated with the standard strains using sterile swabs. After pre-diffusion for 90 min at 4°C, the plates were incubated for 24 h before interpreting the antimicrobial activity results. Finally, positive antimicrobial activity of the actinomycetes was recorded as inhibition zones greater than or equal to 10 mm.

### 2.3. Molecular characterization of bioactive actinomycetes

Five bioactive actinomycetes were identified by 16S rRNA gene sequencing. A pure colony from each isolate was inoculated in ISP2 broth and incubated for 5 days at 30°C, after which 1 ml of each broth was transferred into a sterile Eppendorf tube and centrifuged using a benchtop micro-centrifuge at 12 000 r.p.m. for 30 min. After the removal of the supernatant, the pellets were then used for DNA extraction. A GeneJET Plant Genomic DNA Purification Mini Kit was used to harvest genomic DNA according to the manufacturer's protocol. Finally, DNA concentration and purity were measured using a Nanodrop 2000.

The 16S rRNA genes in actinobacterial genomic DNA were amplified by polymerase chain reaction (PCR) using universal primers: 27F (5′-AGAGTTTGATCCTGGCTCAG-3′) and 1492R (5′-GGTTACCTTGTTACGACTT-3′) [19]. Each 50 µl of PCR reaction contained 25 µl of 2× EF-Taq DNA polymerase (SolGent, Korea), 1 µl of each primer and 5 µl of DNA extract, completed with sterile distilled water for a final volume of 50 µl. PCR amplification was performed using a previously described PCR programme [20]. Gel electrophoresis was employed to confirm success of the PCR amplification by running the PCR products on a 1% (w/v) agarose gel with a 1-Kb reference ladder. The PCR products were then purified using the SolGent PCR purification kit (Daejeon, Korea) [21]. Finally, the PCR products were sent to Macrogen, Korea, for sequencing using an ABI 3730XL DNA Analyzer with BigDye Terminator v. 3.1 Cycle Sequencing Kits (Thermo Fisher Scientific, USA).

The MegaBLAST tool of the National Center for Biotechnology Information (NCBI) was used to compare the 16S rRNA gene sequences of our isolates with those in the GenBank database. Then, 20 sequences that were highly similar to our amplified 16S rRNA genes were subjected to multiple sequence alignment with our sequences, followed by the generation of a phylogenetic tree using the neighbour-joining method [22] with the Mega-X software. Finally, our bioactive actinomycete 16S rRNA gene sequences were deposited in GenBank with the accession numbers MZ027566, MZ027590, MZ027603, MZ027606 and MZ027616 for isolates SH4, SH8, SH10, SH12 and SH13, respectively.

### 2.4. Fermentation and extraction of secondary metabolites

The bioactive isolates were cultured from glycerol stock into starch casein agar and incubated for 7 days at 30°C. Then, a pure colony from each plate was inoculated in 500 ml Erlenmeyer flasks containing 125 ml ISP2 broth and incubated for 7 days at 30°C on a shaker incubator at 160 r.p.m. A 1 : 1 v/v high-performance liquid chromatography- (HPLC)-grade ethyl acetate solvent was subsequently added to the broth, mixed and left overnight before being filtered through a 100 mm filter paper. Next, liquid–liquid partitioning was performed twice between ethyl acetate and broth to extract all natural products. Then, the ethyl acetate was evaporated using a rotary evaporator. Finally, all residues were weighed to determine their dry weight and then were preserved for future work as previously described [23].

### 2.5. Anti-cancer activity of bioactive actinomycetes

The cytotoxicity of the crude extracts of bioactive actinomycetes was assessed using the methyl thiazolyl tetrazolium (MTT) (3-[4,5-dimethylthiazol-2-yl]-2,5-diphenyltetrazolium bromide) assay as described earlier [24,25] with few modifications. The cancer cell lines, human hepatoma HepG2 cells, human breast cancer cell line MCF-7 and corresponding normal cell lines, namely, THLE2 and MCF-10A, were, respectively, purchased from the American Type Culture Collection (ATCC). Cytotoxicity assay was performed at Diagnostic Unit, VACSERA, El-Haram, Giza, Egypt.

Briefly, Dulbecco's modified eagle medium (DMEM) containing 10% fetal bovine serum, 10 µg ml^−1^ insulin and 1% penicillin–streptomycin was used to maintain all cell lines in 96-well plates at 37°C and 5% CO_2_ until they reached a cell density of 1.2–1.8 × 10^4^ cells/well. Both cancer and normal cells were treated with serial concentrations of aqueous solution of different extracts and standard anti-cancer agent in concentration of 100, 25, 6.3, 1.6, 0.4 µg ml^−1^. Then, the 96-well plates were incubated at 37°C and 5% CO_2_ for 48 h before examination under inverted microscope. Untreated cells were used as control for the growth of the cell lines, while culture medium (DMEM) was used as a blank. The cytotoxic activity of different extracts and standard drug was evaluated using MTT assay. The treated and untreated cell lines were incubated with MTT for 2–4 h (depending on cell type and maximum cell density) at 37°C in a CO_2_ incubator. Then, the culture media were aspirated, and the formazan product was solubilized using MTT solubilizing solution, M-8910 (10% Triton X-100 and 0.1 N HCl in anhydrous isopropanol). Finally, MTT absorbance was then quantified using a microtitre plate reader at 570 nm, and multi-well plate background absorbance was recorded spectrophotometrically at 690 nm and subsequently subtracted from the 450 nm reading [25]. For each tumour cell line, the association between viable cells and extract concentration was plotted to determine a survival curve, and IC_50_, which is the concentration of actinomycete extract causing a 50% reduction in absorbance when compared with the control value, was also calculated. The trial was done in triplicates using the standard anti-tumour agent 5-fluorouracil.

### 2.6. Liquid chromatography–high-resolution mass spectrometry

Secondary metabolites in the bioactive crude extracts were chromatographically separated using high-performance liquid chromatography (Accela HPLC, Thermo Fisher Scientific) in conjunction with an Accela UV detector and Exactive (Orbitrap) mass spectrometer (Thermo Fisher Scientific) to determine their exact mass. The HPLC run was performed using an rpHPLC column (BEH C18, 2.1 × 100 mm, 1.7 µm particle size; Waters, USA) protected by a guard column (2.1 × 5 mm, 1.7 µm particle size). The mobile phase consisted of HPLC-grade water containing 0.1% formic acid (v/v) (A) and HPLC-grade acetonitrile (B) with a flow rate of 300 µl min^−1^. The solvent gradient was adjusted to increase from 10% B to 100% B over 30 min and then maintained for 5 min before decreasing back to 10% B for final washing. The column temperature was set to 40°C and the mass detector to both positive and negative ionization modes. In addition, the capillary temperature was set to 270°C with a spray voltage of 4.5 kV, capillary voltage of 35 V and tube lens voltage of 110 V.

### 2.7. Dereplication of liquid chromatography–high-resolution mass spectrometry data

The raw mass data were split into negative and positive datasets using the MassConvert tool from ProteoWizard [26] and imported into the MZmine software in the mzML format [27], in which a multi-step protocol was followed as previously described [15,28]. Then, an in-house Excel macro was written to combine the data files of the negative and positive ionization modes generated by the MZmine software as previously described [15,28]. An in-house macro was also used to dereplicate all mass ion peaks with different natural products for our bioactive isolates in DNP [11,15]. Hits of the known natural products from the database were retrieved using ChemBioFinder version 13 (PerkinElmer Informatics, Cambridge, UK). Then, the actinobacterial hits obtained from DNP were investigated using the free online database of natural products available at http://www.knapsackfamily.com/knapsack_core/top.php.

### 2.8. Metabolomics data analysis of the liquid chromatography–high-resolution mass spectrometry data

The large dataset produced by the metabolomics study required multivariate analysis (MVA) for data interpretation. The liquid chromatography–high-resolution mass spectrometry (LC-HRMS) data of the bioactive isolates were thus analysed and interpreted using MetaboAnalyst 4.0, a web-based statistical analysis program. This investigation used a file comprising a table with sample name, peak list (mass-to-charge ratio; *m/z*) and peak intensities exported as comma-separated values (csv). The MS peak list and intensity data were uploaded as one Zip file to the MetaboAnalyst 4.0 server (https://www.metaboanalyst.ca). Pareto scaling was employed to normalize the data. Then, univariate analysis was conducted using statistical analysis and MVA comprising the principal component analysis (PCA), hierarchical cluster analysis (HCA) and sparse partial least-squares discriminant analysis (sPLS-DA). PCA distinguished the chemical profile of all the samples from each other, whereas sPLS-DA distinguished the metabolites of all samples [29]. A heat map was generated from the LC-HRMS data of all bioactive isolates to show the intensity of significant compounds in the chemical profiles of our crude extracts.

### 2.9. Statistical analysis

Cytotoxicity results in the current study were statistically analysed using Excel. All IC_50_ values were calculated and expressed as mean ± s.d. of the triplicate experiments. The values of IC_50_ of the bacterial extracts in addition to the standard agent, 5-fluorouracil, against the tested cancer cell lines were compared with those against the normal cell lines using unpaired student's *t*-test to calculate the statistical significance. Values of *p* ≤ 0.05 were considered as statistically significant.

## 3. Results and discussion

### 3.1. Antimicrobial activity screening of isolated actinomycetes

Five bioactive isolates (SH4, SH8, SH10, SH12 and SH13) were isolated, cultured in pure colonies and phenotypically identified as actinomycetes according to their colonial morphology, mycelium discoloration and pigment diffusion as well as their microscopical examination. Actinomycetes were previously reported to be prevalent in this soil [30,31].

As can be seen from table 1, four actinomycetes (SH4, SH8, SH10 and SH13) demonstrated broad spectrum of antimicrobial activity against *C. albicans* as well as at least one Gram-positive bacteria and one Gram-negative bacteria. It is noteworthy that isolate SH4 exhibited the broadest spectrum of antimicrobial activity against all the indicator strains tested. As mentioned in many previous reports [14,15,32,33], the soil is considered a rich source of actinomycetes with antimicrobial activities.

**Table 1.** Result of antimicrobial activity of the actinomycetes isolated in the current study against different indicator strains. Positive antimicrobial activity was recorded for any inhibitory zone with diameter of equal to or more than 9 mm.

isolate	*B. subtilis*	*Listeria monocytogenes*	*Sta. aureus*	*E. coli*	*Salmonella enterica*	*C. albicans*
SH4	+	+	+	+	+	+
SH8	+	+	+	+	−	+
SH10	−	−	+	+	−	+
SH12	−	−	+	−	−	+
SH13	−	+	+	+	−	+

### 3.2. Molecular identification of bioactive actinomycetes

**Figure 1.**
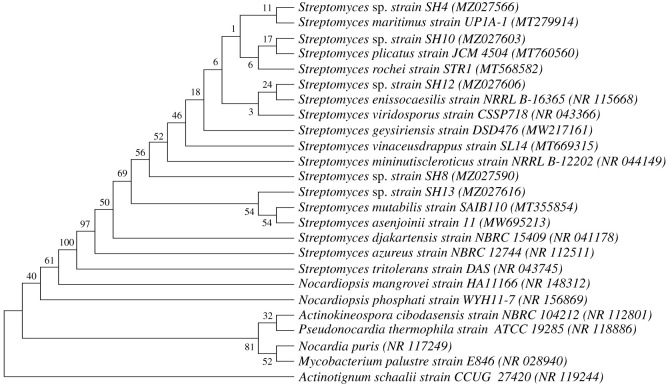
Phylogenetic study of the bioactive *Streptomycetes*' 16S rRNA sequences. The neighbour-joining method was used to create the phylogenetic tree [22]. The evolutionary history of the taxa studied is represented by a bootstrap consensus tree estimated from 1000 replicates [34]. Next to the branches is the percentage of replicate trees in which the associated taxa clustered together in the bootstrap test [34]. The Kimura 2-parameter method was employed to calculate evolutionary distances [35]. A total of 25 nucleotide sequences were examined. 1st + 2nd + 3rd + noncoding codon locations were included. Gaps and missing data were removed from all positions. MEGA-X was used to conduct the tree's evolutionary studies [36].

The homology search using the MegaBLAST tool in NCBI for the 16S rRNA gene sequences of the five bioactive actinomycetes revealed that all their sequences exhibited high similarity (greater than 99%) with several sequences of *Streptomyces* species in the GenBank. Multiple sequence alignment was performed for our sequences with several 16S rRNA gene sequences from NCBI, and a phylogenetic tree was generated using the Mega-X software. As presented in figure 1, all bioactive isolates were assigned as *Streptomyces* sp. considering their closeness to many sequences of different *Streptomyces*, confirming the previous findings from different studies that 16S rRNA is an insufficient tool for assigning exact *Streptomyces* species [4,14,15].

### 3.3. Dereplication of HRMS data

**Figure 2.**
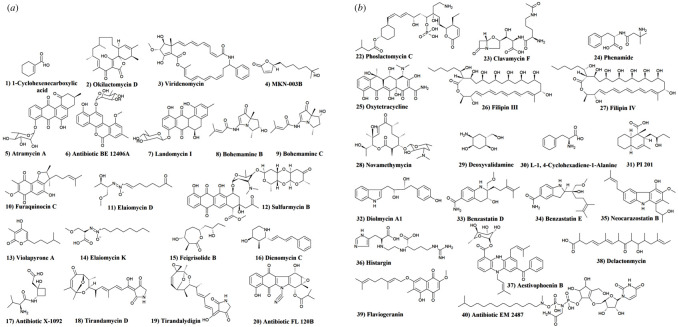
Chemical structure of the closest matches to the metabolites identified from the actinomycetes by molecular weight from databases of natural products (table 2). (*a*) Chemical structure of compounds 1–20. (*b*) Chemical structure of compounds 22–40. The structure of the antibiotic S-583-B (21) is unavailable.

Dereplication of the detected secondary metabolites in the crude extracts can be conducted using available databases, such as DNP [37], and is widely used as a metabolomics tool in natural product discovery for the isolation of new bioactive compounds [11,15,38] and the investigation of their different bioactivities. Herein, a dereplication study was conducted to explore the anti-cancer and antibacterial capabilities of *Streptomycetes* in our study. Dereplication of HRMS data for the bioactive actinomycetes included in our study was performed by comparing their molecular weight and predicted molecular formula (MF) with the known compounds available in the DNP database and online database of natural products available at http://www.knapsackfamily.com/knapsack_core/top.php, to get the closest matches for our detected metabolites. This analysis revealed that most metabolites produced by *Streptomycetes* in our study either had no hits or no actinobacterial hits, indicating a high likelihood of isolation of novel compounds from their crude extracts. In addition, many other secondary metabolites produced by the bioactive *Streptomycetes* in our study were putatively identified as natural products previously isolated from other *Streptomycetes* (table 2 and figure 2). Interestingly, several compounds from these putatively identified metabolites exhibited diverse biological activities, including anti-tumour, antifungal and antibacterial activities, which may explain the antimicrobial and anti-cancer activities of the bioactive *Streptomycetes* in our study.

**Table 2.** Secondary metabolites from the crude extracts of bioactive *Streptomyces* isolates which were dereplicated according to the closest match from natural products' databases (*m/z*, mass/charge ratio, Rt, retention time, MW, molecular weight, MF, molecular formula).

compound	*m/z* (ionization)	Rt (min)	MW	MF	putative identification	source (organism)	references
1	127.0755 [M + H]^+^	2.19	126.0683	C_7_H_10_O_2_	**1-Cyclohexenecarboxylic acid**	*Str. collinus* TU 1892	[39]
2	371.2221 [M − H]^−^	4.94	372.2294	C_23_H_32_O_4_	**Okilactomycin D**	*Str. scabrisporus* F-117187	[40]
3	554.2564 [M − H]^−^	9.42	555.2637	C_34_H_37_NO_6_	**Viridenomycin**	*Str. viridochromogenes* T-24146 and *Str. gannmycicus*	[41,42]
4	213.1483 [M + H]^+^	2.66	212.14099	C_12_H_20_O_3_	**MKN-003B**	*Streptoverticillium luteoverticillatum* 11014 and *Streptomyces* sp. M02750	[43,44]
5	467.1361 [M − H]^−^	1.40	468.1434	C_25_H_24_O_9_	**Atramycin A**	*Str. atratus* BY90	[45]
6	467.1361 [M − H]^−^	1.40	468.1434	C_25_H_24_O_9_	**Antibiotic BE 12406A**	*Streptomyces* sp. BA12406	[46,47]
7	467.13614 [M − H]^−^	1.40	468.1434	C_25_H_24_O_9_	**Landomycin I (8-D-Olivosyl-landomycin)**	*Str. cyanogenus* S-136	[48]
8	265.1546 [M + H]^+^	2.85	264.1473	C_14_H_20_N_2_O_3_	**Bohemamine B**	*Streptomyces* sp. CNQ-583	[49]
9	265.1546 [M + H]^+^	2.85	264.1473	C_14_H_20_N_2_O_3_	**Bohemamine C**	*Streptomyces* sp. CNQ-583	[49]
10	369.1704 [M − H]^−^	2.49	370.1777	C_22_H_26_O_5_	**Furaquinocin C**	*Streptomyces* sp. KO-3988	[50]
11	273.1817 [M + H]^+^	2.52	272.1744	C_13_H_24_N_2_O_4_	**Elaiomycin D**	*Streptomyces* sp. HKI0708	[51]
12	836.3112 [M − H]^−^	6.24	837.3184	C_43_H_51_NO_16_	**Sulfurmycin B**	*Str. galilaeus* OB-111	[52,53]
13	211.1324 [M + H]^+^	4.47	210.1251	C_12_H_18_O_3_	**Violapyrone A**	*Str. violascens* YIM 100525	[54]
14	261.1807 [M + H]^+^	2.13	260.1734	C_12_H_24_N_2_O_4_	**Elaiomycin L**	*Streptomyces* sp. Tü 6399	[55]
15	217.1428 [M + H]^+^	2.87	216.1355	C_11_H_20_O_4_	**Feigrisolide B**	*Str. griseus* GT 051022	[56]
16	244.1695 [M + H]^+^	1.73	243.1623	C_16_H_21_NO	**Dienomycin C**	*Streptomyces* sp. MC67-C1	[57]
17	245.1494 [M + H]^+^	1.81	244.1422	C_11_H_20_N_2_O_4_	**Antibiotic X-1092**	*Streptomyces* sp. X-1092	[58]
18	400.1764 [M − H]^−^	6.76	401.1837	C_22_H_27_NO_6_	**Tirandamycin D**	*Streptomyces* sp. 307–9	[59]
19	400.1764 [M − H]^−^	6.76	401.1837	C_22_H_27_NO_6_	**Tirandalydigin**	*Str. tirandis* subsp. *umidus* AB1006A-9	[60]
20	421.1038 [M − H]^−^	5.59	422.1111	C_22_H_18_N_2_O_7_	**Antibiotic FL 120B**	*Str. chattanoogensis* subsp. *taitungensis* subsp. nov. IY2–13	[61]
21	1135.5238 [M + H]^+^	0.82	1134.5165	C_59_H_78_N_2_O_20_	**Antibiotic S-583-B**	*Str. purpurascens* S-583	[62]
22	614.3094 [M + H]^+^	7.44	613.3021	C_30_H_48_NO_10_P	**Phoslactomycin C**	*Str. nigrescens* SC-273	[63]
23	371.1562 [M − H]^−^	6.53	372.1635	C_15_H_24_N_4_O_7_	**Clavamycin F**	*Str. hygroscopicus* NRRL 15879	[64,65]
24	265.1546 [M + H]^+^	2.93	264.1471	C_14_H_20_N_2_O_3_	**Phenamide**	*Str. albospinus* A19301	[66]
25	459.14032 [M − H]^−^	5.45	460.1476	C_22_H_24_N_2_O_9_	**Oxytetracycline**	*Str. rimosus*	[67,68]
26	655.4051 [M + H]^+^	2.24	654.3978	C_35_H_58_O_11_	**Filipin III**	*Str. filipinensis*	[69,70]
27	655.4051 [M + H]^+^	2.24	654.3978	C_35_H_58_O_11_	**Filipin IV**	*Str. filipinensis*	[69,70]
28	486.3055 [M + H]^+^	3.98	485.2982	C_25_H_43_NO_8_	**Novamethymycin**	*Str. venezuelae*	[71]
29	162.1125 [M + H]^+^	2.82	161.1052	C_7_H_15_NO_3_	**Deoxyvalidamine**	*Str. hygroscopicus* subsp. *Limoneus*	[72]
30	168.1023 [M + H]^+^	3.64	167.095	C_9_H_13_NO_2_	**L-1, 4-Cyclohexadiene-1-alanine**	*Str. diastatochromogenes* var. *sakaii.*	[73]
31	281.2106 [M + H]^+^	2.00	280.2033	C_17_H_28_O_3_	**PI 201**	*Streptomyces* sp. A7498	[74]
32	298.1434 [M + H]^+^	4.43	297.1361	C_18_H_19_NO_3_	**Diolmycin A1**	*Streptomyces* sp. WK-2955	[75]
33	331.2018 [M − H]^−^	1.80	332.2091	C_19_H_28_N_2_O_3_	**Benzastatine D**	*Str. nitrosporeus* 30643	[76]
34	331.2018 [M − H]^−^	1.80	332.2091	C_19_H_28_N_2_O_3_	**Benzastatine E**	*Str. nitrosporeus* 30643	[77]
35	352.1921 [M − H]^−^	6.82	353.1994	C_22_H_27_NO_3_	**Neocarazostatin B**	*Streptomyces* sp. GP38	[78]
36	354.1902 [M − H]^−^	6.93	355.1975	C_14_H_25_N_7_O_4_	**Histargin**	*Str. roseoviridis* MF118-A5	[79]
37	613.2894 [M + H]^+^	0.47	612.2821	C_36_H_40_N_2_O_7_	**Aestivophoenin B**	*Str. purpeofuscus* 2887-SVS2	[80]
38	365.2696 [M + H]^+^	1.86	364.2624	C_22_H_36_O_4_	**Delactonmycin**	*Streptomyces* sp. A92–308902	[81]
39	369.1704 [M + H]^+^	2.49	370.1777	C_22_H_26_O_5_	**Flaviogeranin**	*Streptomyces* sp. RAC226	[82]
40	828.3187 [M − H]^−^	4.87	829.3259	C_32_H_57_N_5_O_16_P_2_	**Antibiotic EM 2487**	*Streptomyces* sp. Mer-2487	[83]

Several secondary metabolites produced by the bioactive isolates examined in the current study were dereplicated according to the closest matches from the screened databases as cytotoxic compounds previously extracted from *Streptomycetes*. For instance, the mass ion peak at *m/z* 554.2564 [M − H]^−^ with a predicted MF C_34_H_37_NO_6_ had the predicted match of **Viridenomycin** (**3**), a polyene lactam antibiotic isolated from *Str. gannmycicus* exhibiting anti-tumour activity against murine tumours (ionic liquid toxicity of 23% for P388 leukaemia and 37% for B16 melanoma) [41]. **Viridenomycin** was also previously extracted from *Str. viridochromogenes* T-24146 and was found to display potent antibacterial activity against *Sta. aureus* with a minimum inhibitory concentration (MIC) value below 1 µg ml^−1^ and *B. subtilis* with an MIC value of 0.05 µg ml^−1^. It also exhibited antiprotozoal bioactivity against *Trichomonas vaginalis* with an MIC value of 0.06 µg ml^−1^ [42]. Moreover, the mass ion peak at *m/z* 213.1483 [M + H]^+^ with predicted MF C_12_H_20_O_3_ was chemically annotated as the small natural product **MKN-003B** (**4**), previously isolated from the marine actinomycete *Streptoverticillium luteoverticillatum* 11014 and displaying anti-tumour activity against the human leukaemia K562 and murine lymphoma P388 cell lines with IC_50_ values of 8.73 and 0.34 µM, respectively [43]. **MKN-004B** was also isolated earlier from *Streptomyces* sp. M02750 and was found to exhibit antifungal activity against *C. albicans* [44].

In addition, the mass ion peak at *m/z* 467.13614 [M − H]^−^ with predicted MF C_25_H_24_O_9_ was dereplicated according to the closest match from databases to be the anthraquinone derivative **Atramycin A** (**5**), previously isolated from *Str. atratus* BY90 and demonstrating anti-tumour properties against P388 leukaemia cells with an IC_50_ value of 4.5 µg ml^−1^ [45], and as the antibiotic **BE 12406A** (**6**), extracted from *Streptomyces* sp. BA12408 and exhibiting cytotoxic activity against P388 murine leukaemia with an IC_50_ value of 0.2 µM [46]. This mass ion peak was also putatively identified as the polyketide natural product **Landomycin I** (**7**) isolated from *Str. cyanogenus* S-136 with selected potent cytotoxicity against the murine Lewis lung cancer LL/2 and human MCF-7 cell lines with growth inhibitory 50% (IG_50_) values of 3.5 and 3.7 µg ml^−1^, respectively [48].

Furthermore, several other secondary metabolites detected in our crude extracts were chemically annotated as bioactive antimicrobial compounds previously produced by *Streptomycetes* when compared with databases to get the closest predicted matches. For example, the mass ion peak at *m/z* 211.1324 [M + H]^+^ with predicted MF C_12_H_18_O_3_ was putatively identified as **Violapyrone A** (**13**) produced by *Str. violascens* YIM 100525 and exhibiting moderate antibacterial activities against *Sta. aureus* and *B. subtilis* with MIC values of 8 and 32 µg ml^−1^, respectively [54]. The mass ion peak at *m/z* 261.1807 [M + H]^+^ with predicted MF C_12_H_24_N_2_O_4_ was annotated as **Elaiomycin L** (**14**), separated from *Streptomyces* sp. Tü 6399 and displaying antibacterial activity against *B. subtilis*, *Sta. lentus* and *Xanthomonas campestris* with IC_50_ values of 22.9, 41.7 and 51.3 µM, respectively [55]. The mass ion peak at *m/z* 217.1428 [M + H]^+^ with predicted MF C_11_H_20_O_4_ was dereplicated according to the closest match from databases to be **Feigrisolide B** (**15**), isolated from *Str. griseus* GT 051022 and exhibiting moderate antifungal activity against *Sporobolomyces salmonicolor*, as well as moderate antiviral activity against the Coxsackie virus [56]. It is noteworthy that the mass ion peak at *m/z* 400.1764 [M − H]^−^ with predicted MF C_22_H_27_NO_6_ was chemically annotated as the tetramic acid derivative **Tirandamycin D** (**18**), isolated from *Streptomyces* sp. 307–9 and demonstrating good bioactivity against the vancomycin-resistant *Enterococcus faecalis* with an MIC value of 9 µM [59]. This mass ion peak was also annotated as the other tetramic acid derivative, **Tirandalydigin** (**19**), isolated from *S.tr tirandis* subsp. *umidus* AB1006A-9 and showing antibacterial activity against several pathogens, including *Peptostreptococcus anaerobius*, *Clostridium perfringens*, *Bacteroides thetaiotaomicron*, *P. magnus* and *C. difficile* with MIC values of 0.06, 0.25, 0.5, 16 and 32 µg ml^−1^, respectively [60].

Moreover, the mass ion peak at *m/z* 421.1038 [M − H]^−^ with predicted MF C_22_H_18_N_2_O_7_ was annotated as the polyketide natural antibiotic **FL 120B** (**20**), which is an epoxide derivative of kinamycin antibiotic. **FL 120B** was previously reported from *Str. chattanoogensis* subsp. *taitungensis* IY2–13 and has potent antibacterial activity against many microbes, including *B. anthracis*, *Neisseria gonorrhoeae*, *C. tetani*, *Sta. aureus* and *Streptococcus pneumoniae* with MIC values of 0.01, 0.02, 0.2, 0.4 and 0.78 µg ml^−1^, respectively [61]. The mass ion peak at *m/z* 614.3094 [M + H]^+^ with predicted MF C_30_H_48_NO_10_P was putatively identified as **Phoslactomycin C** (**22**), previously isolated from *Str. nigrescens* SC-273 and displaying antifungal activities against *Cladosporium fulvum* (inhibition zone = 25 mm), *Ustilago maydis* (inhibition zone = 15 mm) and *Pyricularia oryzae* (inhibition zone = 12 mm) [63]. Moreover, the mass ion peak at *m/z* 371.1562 [M − H]^−^ with predicted MF C_15_H_24_N_4_O_7_ was chemically annotated as the clavam antibiotic **Clavamycin F** (**23**), isolated from *Str. hygroscopicus* NRRL 15879 and possessing antifungal properties [64,65]. It is noteworthy that two other mass ion peaks at *m/z* 459.14032 [M − H]^−^ with predicted MF C_22_H_24_N_2_O_9_ and *m/z* 486.3055 [M + H]^+^ with predicted MF C_25_H_43_NO_8_ were putatively identified as the tetracycline antibiotic **Oxytetracyclin**e (**25**), previously isolated from *Str. rimosus* [67,68], and the macrolide antibiotic **Novamethymycin** (**28**), isolated earlier from *Str. venezuelae* [71].

### 3.4. Anti-cancer activity of *Streptomycetes*

**Figure 3.**
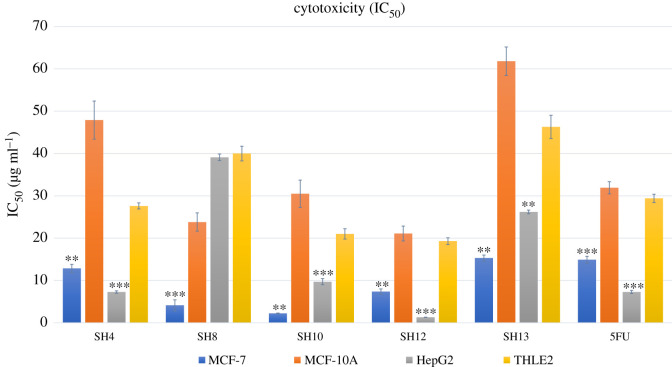
Anti-cancer activity of the bioactive *Streptomycetes* against the cancer cell lines MCF-7 and HepG2 as well as their normal cell lines MCF-10A and THLE2, respectively. 5FU = 5-fluorouracil. The cytotoxicity of the crude extracts and 5-fluorouracil drug was evaluated using MTT assay in MCF-7, MCF-10A, HepG2 and THLE2 cell lines upon 48 h treatment with different concentrations of bacterial extracts and standard drug. Values of IC_50_ were presented as mean ± s.d., while statistical significance of anti-cancer activity of different extracts and 5-fluorouracil against tested cancer cell lines comparing with normal cell lines was calculated using unpaired student's *t*-test where *p < 0.05,**p < 0.01 and ***p < 0.001. The experiment was performed in triplicates.

Scientists continue to search for natural sources of anti-cancer agents, which have helped in discovering new drugs for managing tumours [10]. Our crude extracts were tested for their anti-cancer and cytotoxic effects against two cancer cell lines, HepG2 and MCF-7, and their normal cell lines, THLE2 and MCF-10A, respectively. All the tested *Streptomycetes* demonstrated positive anti-tumour activity against MCF-7, with weak inhibitory activity against MCF-10A (figure 3). Of note, the isolates SH10, SH8 and SH12 exhibited potent anti-cancer activity against MCF-7, with IC_50_ values of 2.22, 4.12 and 7.37 µg ml^−1^, respectively (figure 3). Contrarily, the isolates SH12, SH4 and SH10 demonstrated strong anti-cancer activity against the HepG2 cell line, with IC_50_ values of 1.31, 7.27 and 9.7 µg ml^−1^, respectively, compared with their inhibitory activities on the liver normal cell line THLE2 (figure 3). Interestingly, the IC_50_ values of potent bioactive extracts were either equivalent or superior to the anti-cancer drug 5-fluorouracil, which showed IC_50_ values of 14.9 and 7.28 µg ml^−1^ against MCF-7 and HepG2, respectively.

Our extracts’ cytotoxicity assay results were consistent with those of several previously published data [10,84]. Numerous anti-tumour compounds were isolated earlier from various species of *Streptomyces*, including 1-(1H-indol-3-yl)-propane-1,2,3-triol and Chromomycin SA isolated from *Streptomyces* sp. KML-2 with significant anti-tumour activity against the MCF-7 and HeLa cancer cell lines, with IC_50_ values of 0.97 and 12.6 µg ml^−1^ against MCF-7 and, 7.8 and 8.9 µg ml^−1^ against HeLa, respectively [85]. Also, benzoxazole isolated from soil *Streptomyces* sp. Tü 6176 displayed potent anti-tumour activity against the AGS (gastric adenocarcinoma), MCF-7 and HepG2 cell lines, with IC_50_ values of 0.4, 0.68 and 0.06 µg ml^−1^, respectively [86]. In addition, di-(2-ethylhexyl) phthalate isolated from *Str. mirabilis* NSQu-25 exhibited anti-tumour activity against MCF-7, HepG2 and the human colon carcinoma cell line HCT 116, with IC_50_ values of 6.941, 9.028 and 3.681 µg ml^−1^, respectively [17].

### 3.5. Metabolomic profiling of the bioactive isolates

Metabolomics involves a multi-step protocol including sample preparation, instrumental analysis using tools such as LC-MS or NMR, followed by data processing and clean-up, and finally data analysis and interpretation. LC-MS and especially LC-HRMS are commonly used for instrumental analysis of samples prior to data analysis [29,37]. Dereplication and MVA are usually employed together as they constitute an excellent approach for drug discovery [11,38,87]. MVA is one of the chemometric tools for the analysis and interpretation of metabolomics data. Both unsupervised and supervised MVA are widely used for investigating variation among various sample groups in terms of *m/z* ratio or chemical shifts (ppm) in LC-HRMS and NMR, respectively [88].

**Figure 4.**
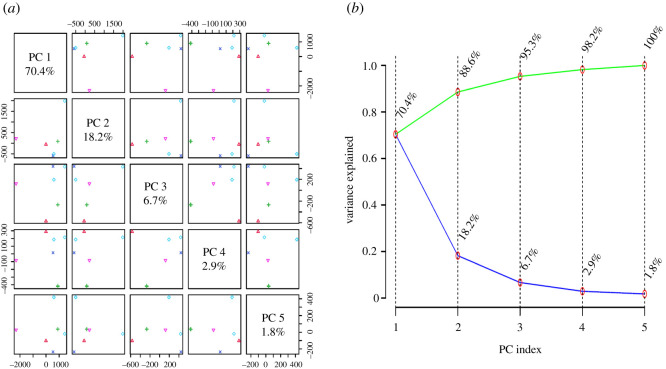
The contribution of different principal components in determining the variation of the PCA model in the current study. (*a*) Pairwise PCA score plot of different principal components in the unsupervised model. (*b*) PCA scree plot of different principal components in the unsupervised model.

In the current study, MVA was performed using MetaboAnalyst 4.0 to minimize the dataset size from LC-HRMS analysis, correlate the findings and present the final conclusions [37,89]. First, we implemented an unsupervised PCA method for clustering of LC-HRMS data generated for the five bioactive extracts considering its capability to decrease the MVA dimensions and investigate the chemical variation between metabolomic profiles without previous knowledge of the dataset [89]. With our PCA model, *R*^2^ (goodness of fitness) and *Q*^2^ (predictability) values were higher than 0.8 indicating a good performance, considering *R*^2^ and *Q*^2^ as application-dependent [90] and a threshold of significance for the *Q*^2^ parameter of 0.5 [91]. As presented in figure 4, there are five total principal components from which PC1, PC2 and PC3 explained 95.3% of the total variation in the PCA model, whereas PC1 and PC2 contributed to 88.6% of the total variation in PCA.

**Figure 5.**
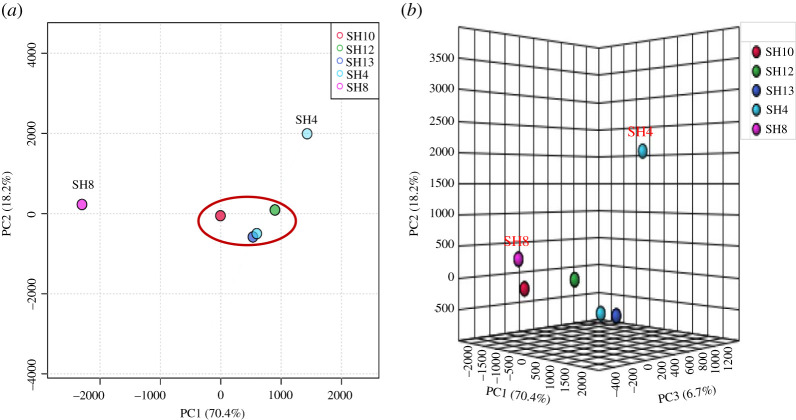
Principal component analysis of bioactive actinobacterial extracts. (*a*) Two-dimensional PCA score plot. (*b*) Three-dimensional PCA score plot.

**Figure 6.**
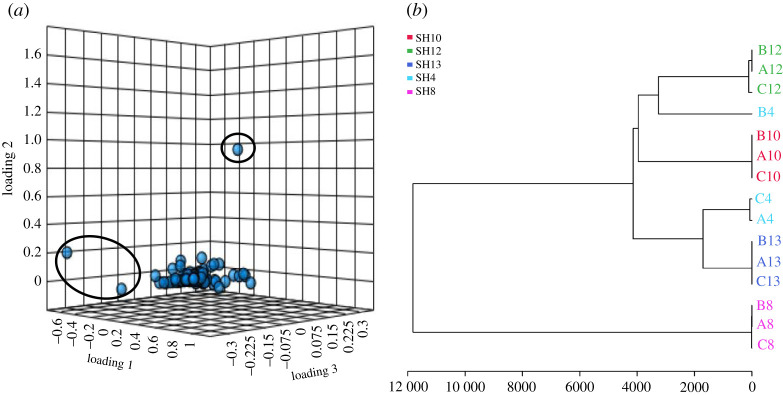
Principal component analysis loadings plot and hierarchical cluster analysis of bioactive actinobacterial extracts. (*a*) Three-dimensional PCA loadings plot with discriminatory compounds to SH4, SH8 and SH10. (*b*) HCA plot.

As presented in figure 5*a,b*, PCA revealed the chemical variation between different metabolomic profiles of isolates regardless of their bioactivity. The two-dimensional PCA score plot for PC1 and PC2 displays the extracts of SH4 and SH8 with a different chemical profile compared with other extracts (SH10, SH12, and SH13) clustering close to each other. However, the three-dimensional PCA score plot for PC1, PC2 and PC3 shows scattered pattern of extract chemical profiles, whereas the metabolomic profile of SH4 was farther from other actinobacterial profiles (figure 5*b*). As presented in figure 6*a*, the two-dimensional loading plot of top 50 features identified by one-way analysis of variance and *post hoc* analysis highlights the metabolites (*m/z*) standing behind the chemical variation between crude extracts, as presented in the score plot. It is noteworthy that three main compounds were identified as discriminatory metabolites for SH4, SH8 and SH10 in the loading plot, with mass ion peaks at *m/z* 304.3651 [M + H]^+^, *m/z* 332.4022 [M + H]^+^ and *m/z* 340.2236 [M − H]^−^ (figure 6*a*). Only the mass ion peak at *m/z* 340.2236 [M − H]^−^ with predicted MF C_17_H_31_N_3_O_4_ was dereplicated according to the closest match from databases to be **Diprotin A**, a small peptide isolated earlier from *B. cereus* as an inhibitor of dipeptidyl aminopeptidase IV [92]. Moreover, HCA was employed to show good visualization of chemical variation between crude extracts to facilitate dataset analysis using other MVA tools [29]. Here, the HCA plot was presented as a dendrogram showing two main clusters of metabolomic profiles between extracts (figure 6*b*). The first cluster only contained isolate SH8, indicating its chemical uniqueness in accordance with the PCA results, whereas the second cluster comprised all the other isolates. The second clustering group was further separated into two primary groups, with SH4 and SH13 in group 1 and SH10 and SH12 in group 2 (figure 6*b*).

**Figure 7.**
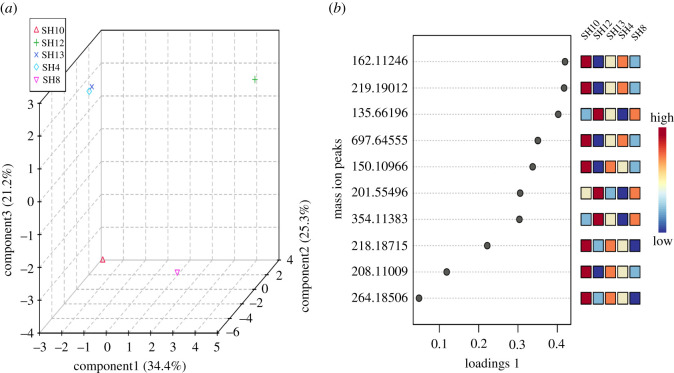
Supervised multivariate analysis of bioactive actinobacterial extracts. (*a*) Three-dimensional sPLS-DA score plot. (*b*) VIP score plot.

To generate robust models, the supervised sPLS-DA algorithm was used to minimize the number of metabolites in high-dimensional metabolomics data. In our sPLS-DA model, three PLS components represented 80.9% of the total variation in the model. The first PLS component contributed to 34.4% of the total variation, whereas the second and third PLS components provided 25.3% and 21.2% of the total variation, respectively (figure 7*a*). As presented in figure 7*a*, chemical profiles of bioactive *Streptomycetes* in the three-dimensional score plot of sPLS-DA were shown in a scattered manner except for SH4 and SH13, which were close to each other. Also, SH4, SH12 and SH13 were far from SH8 and SH10 in the score plot.

The variable importance in projection (VIP) was next used to distinguish crude extracts by taking into account their most important features with the highest value indicated by sPLS-DA [29]. In the VIP plot, the 10 most important metabolites identified by sPLS-DA are presented in ascending manner based on their intensity in bioactive extracts (figure 7*b*). Dereplication of VIP metabolites using DNP revealed that only one metabolite was putatively identified as a known natural product, whereas the other nine ion peaks were related to unknown compounds. The mass ion peak *m/z* 162.1125 [M + H]^+^ corresponding to the predicted MF C_7_H_15_NO_3_ was dereplicated according to the closest match from databases to be as **Deoxyvalidamine** (**29**), which was previously isolated from *Str. hygroscopicus* subsp. *limoneus* as a glucosidase inhibitor [72].

**Figure 8.**
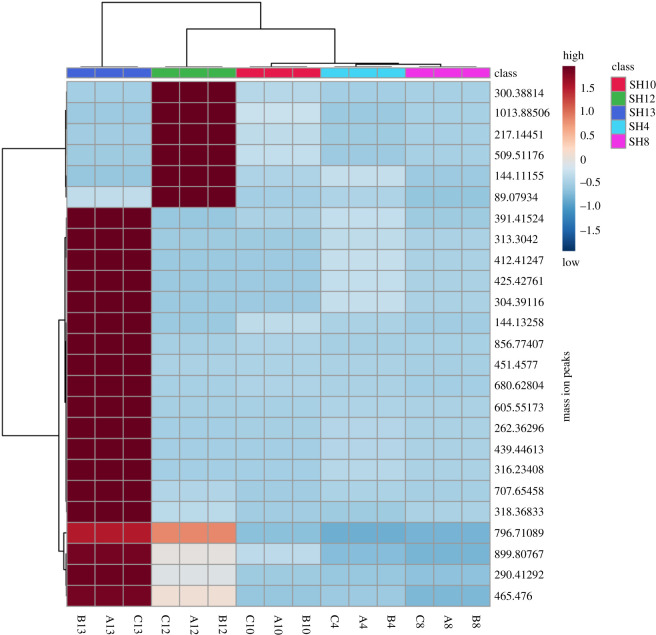
Clustering of crude extracts from bioactive *Streptomycetes* according to the intensity of mass ion peaks of main metabolites as shown in the heat map. Red indicator is for high intensity of mass ion peaks, while blue is indicating lower intensity of mass ion peaks in the clustered crude extracts.

In our study, the heat map showed the distribution of the main metabolites present in the active *Streptomycetes* extracts (figure 8). SH12 and SH13 were the most chemically different extracts among the actinobacterial isolates based on the intensity of significant metabolites partially matching the result of the sPLS-DA 3D score plot (figure 7*a*). In addition, both SH12 and SH13 were shown to be chemically different in terms of the intensity of metabolites in crude extracts, supporting the result of HCA grouping them in the same cluster under different groups (figure 6*b*).

The results of MVA suggested that the chemical profiles of the bioactive *Streptomycetes* were quite different from each other, indicating richness of their metabolomes in terms of interesting secondary metabolites. This was clear especially when implementing HCA, sPLS-DA and heat map, which supports the idea that SH12 and SH13 have unique metabolites regardless of their different intensities in SH12 and SH13 presented in the heat map and confirmed by HCA. SH4 was also shown by both PCA and sPLS-DA to have a distinct chemical profile, indicating its richness of antibacterial and anti-cancer metabolites. SH4 also demonstrated the broadest antimicrobial activity against all the tested indicator microorganisms, with a robust activity against both the HepG2 and MCF-7 cell lines. Furthermore, the dereplication metabolomics analysis of putatively identified secondary metabolites helped in understanding the antimicrobial and anti-tumour activities of the crude extracts by identifying several of its bioactive compounds with diverse activities previously isolated from *Streptomycetes*.

## 4. Conclusion

The current study highlights the importance of combining dereplication and metabolomics data analysis, including the use of supervised and unsupervised MVA, when chemically profiling *Streptomycetes* for isolate prioritization and chemical isolation. Implementing a dereplication- and multivariate analysis-based approach could also help in understanding how secondary metabolites underlie different biological activities. Also, conducting a metabolomics-guided approach supported by the results of dereplication and different bioassays could increase the likelihood of targeting and hence isolating new bioactive natural products from different sources, including *Streptomycetes*. In addition, we can see that combining metabolomics bioinformatic analysis with other databases, such as antiSMASH, which can assign the different gene clusters encoding secondary metabolites' production, could increase the chance of natural products’ discovery. Moreover, it was concluded in our study that the Egyptian soil actinomycetes remain a viable source for isolating novel bioactive natural compounds, particularly using a metabolomics-based approach instead of traditional bioassay-guided techniques.

## Data accessibility

The 16S rRNA gene sequences of our isolates were deposited in NCBI GenBank with the accession nos. MZ027566, MZ027590, MZ027603, MZ027606 and MZ027616 for isolates SH4, SH8, SH10, SH12 and SH13, respectively.
